# Knowledge on Hypertension and Self-Care Practice among Adult Hypertensive Patients at University of Gondar Comprehensive Specialized Hospital, Ethiopia, 2019

**DOI:** 10.1155/2020/5649165

**Published:** 2020-04-20

**Authors:** Chanyalew Worku Kassahun, Ayele Asasahegn, Desalegn Hagos, Elshaday Ashenafi, Firegenet Tamene, Getachew Addis, Kidist Endalkachew

**Affiliations:** Department of Comprehensive Nursing, College of Medicine and Health Sciences, University of Gondar, Gondar, Ethiopia

## Abstract

**Background:**

Patients with hypertension need to be aware of various aspects of hypertension and exercise self-care. But, there is limited information regarding this issue. *Objective of the Study*. This study was designed to assess knowledge on hypertension and self-care practice among adult hypertensive patients in the University of Gondar Comprehensive Specialized Hospital, Ethiopia.

**Materials and Methods:**

Descriptive cross-sectional study was conducted among 384 hypertensive patients from April to May 2019. The study participants were selected using a systematic random sampling technique. Data were collected using a pretested interviewer-administered questionnaire. Descriptive statistics such as percentage and frequency of patients' knowledge on hypertension and their self-care practice were computed. Cross-tabulation was used to see the frequency and percentage of selected sociodemographic variables and knowledge level with self-care practice subscales. Finally, the results were summarized and presented in texts, figures, and tables.

**Results:**

Among the study participants, 215 (56%) and 228 (59.4%) had good knowledge and self-care practice towards hypertension, respectively. The participants who had good knowledge had good self-care practice frequency.

**Conclusions:**

In this study, knowledge on hypertension was low, while self-care practice was moderate on the self-care interventions. Hence, increasing patients' awareness and intervention on medication adherence, low salt diet consumption, physical activity, weight management, cigarette smoking cessation, and alcohol consumption reduction is important.

## 1. Background

More than 80% of the burden of hypertension in low-income and middle-income countries is because of the lack of information and poor self-care practice [1]. Lack of knowledge about hypertension is a major challenge in controlling hypertension. To reduce this burden, patients have to be counseled on lifestyle changes when they visit their health facility and take measures regarding self-care [2–4]_._ The self-care involves medication adherence, eating low-fat diet, regular physical exercise, limiting alcohol consumption, not smoking, weight reduction, self-monitoring of blood pressure (BP), regular health care visit, and reducing stress [5–10].

In a study conducted in Saudi Arabia, around 68% of patients knew their target BP level [11], while around 70% of patients did not know their BP level in Asia [12]. In South India, 52.4% of hypertensive patients had average or good knowledge [13], and 14%–56% were aware of hypertension in Nepal [14]. On the other hand, 82% of patients did not know about hypertension and 92.2% had inadequate knowledge on hypertension in Canada and Sri Lanka, respectively [15, 16]. In Botswana, more than 96% of patients responded as reducing smoking and stress can prevent hypertension, but almost equal proportion of participants were knowledgeable about alcohol restriction (65.2%), dietary restriction (66.3%), and physical exercise [17]. In Addis Ababa, Ethiopia, 43.6% hypertensive patients had good knowledge about hypertension [18], 9.2% knew the ideal BP, and 67.7% believed that exercise can reduce BP in a study conducted in Jimma University [19].

In India, 62.9% of the study participants had unfavorable self-care practice [20]. But, in Jeddah, Saudi Arabia, the self-care practice ranged from 31.2% to 83.7% in each self-care subscales [11]. In a study conducted in Western Nepal, more than 55% of patients involved in their own care in each self-care practice components (70% did not take alcohol/quit smoking, 80.6% took low fat and salt diet, 69.7% monitored their BP regularly, 58.2% reduced their stress, and 85% used medication regularly) [21]. In another study conducted in outreach clinic of South India indicated that 11.4%, 49.2%, and 39.2% hypertensive patients had good, average, and poor self-care practice, respectively [13]. A study conducted in Northwestern Nigeria showed that majority of the participants had inadequate self-care practice [22]. In studies conducted in Ethiopia, 51% of hypertension patients had poor self-care practice in Dessie town [23], and 51.5% of study participants had good self-care practice in Addis Ababa [18]. In addition, only 1.5% of participants smoked and 94.6% followed salt restrictions in Jimma University [19].

Nevertheless, limited studies have been conducted on this issue in Ethiopia, in the study area in particular. Therefore, this study is designed to assess knowledge on hypertension and self-care practice among adult hypertensive patients.

## 2. Materials and Methods

### 2.1. Study Design, Area, and Period

This hospital-based descriptive cross-sectional study design was conducted at the University of Gondar Comprehensive Specialized Hospital (UoGCSH) from April to May 2019. UoGCSH provides different specialized services in four major departments: the Pediatrics, Surgery, Gynecology and Obstetrics, and Internal Medicine. It also has its own chronic disease such as hypertensive patients follow-up service. Hypertensive patients are attending their follow-up monthly. On average, 450 adult hypertensive patients are attending their follow-up in the hospital per month [24].

### 2.2. Study Population

All adult hypertensive patients on follow-up were the source population. Hypertensive patients available during data collection period were included. Participants who have attended their follow-up less than six months and could not to be interviewed were excluded.

## 3. Sample Size, Sample Technique, and Procedure

The sample size was calculated by using a single population proportion formula:

(1) $$\begin{array}{c} n=\frac{\left(z^{2}\right)\left(p\right)\left(1-p\right)}{d^{2}}, \end{array}$$

where *n* = sample size, *z* = 1.96 at 95% confidence level, *d* = 5% margin of error, and *p*=51.5%, proportion for self-care practice among hypertensive patient, which was taken from a study conducted at Addis Ababa [18]. Accordingly, the sample size was 384. The study used systematic random sampling technique. Card numbers of the study participants were selected first from the log book according to their follow-up. Next, patients were selected using eligibility criteria. Then, unique numbers were given for each participant. The sampling frame was determined from the monthly patient flow, i.e., every two patients were used to select each study participants. The first person was selected by simple random sampling.

### 3.1. Operational Definitions

#### 3.1.1. Knowledge

Participants were labeled as knowledgeable if they scored median and above and nonknowledgeable if they scored below the median. Twelve items were used to measure knowledge. The response of participants in each item was coded as “Yes” if they answered the items correctly, and “No” if they answered incorrectly. Median was calculated from items which were answered correctly.

#### 3.1.2. Self-Care Practice

Patients report on their engagement with any kind of recommended self-care activities. Participants were categorized as having good practice if they scored median and above and having poor practice if they scored below the median [25]. Self-care practice was measured by Hypertension Self-Care Activity Level Scale Effects (H-SCALE), which has six outcome domains: medication adherence (3 items): responses range from 0 to 21, and participants who reported that they followed the 3 recommendations on 7 out of 7 days were considered adherent (score = 21). Low-salt diet (12 items): scores of 6 out of 7 days were considered adherent. Physical activity (2 items): responses range from 0 to 14. Participants who scored 8 or better were considered adherent. Smoking (2 items): responses ranges from 0 to 14, and respondents who reported 0 days were considered a nonsmoker and all others were smokers. Weight management (10 items): responses range from 10 to 50. Participants who agreed or strongly agreed with all 10 items (score ≥40) were considered to be following good weight management practices. Alcohol (3 items): responses range from 0 to 21. Participants who did not take any alcohol in the last 7 days or who did not drink at all were considered abstainers. All others were nonadherent.

### 3.2. Data Collection Instrument/Tools and Procedure

Semistructured interviewer-administered questionnaire was used to collect the data. The tool has three sections: Section I: sociodemographic information; Section II: knowledge-related questions; Section III: self-care-related questions. Knowledge on hypertension was assessed by a validated tool for hypertensive patients [26]. To measure self-care practice, Hypertension Self-Care Activity Level Effects (H-SCALE) which was used in a previous study in Jimma, Ethiopia, was used [27]. All questionnaires were prepared in English language and then translated to Amharic (local language), which has been used for data collection. To keep the quality of the data completeness of the questionnaire, it was checked before data entry, and EPI-data version 3.1 was used to minimize the data entry error.

### 3.3. Data Processing and Analysis

Data were checked for completeness, edited, and entered to EPI-data version 3.1 and exported to SPSS version 21 for analysis. The data were explored to see missing values, the shape, and distribution of the data. On the basis of this information, there are no missing values, and the data were almost normally distributed. Descriptive statistics (frequencies, percentages, mean values, and standard deviations) were calculated for demographic characteristics. Then, the percentage and frequency of patients' knowledge on hypertension and their self-care practice were computed. Cross-tabulation was used to check the frequency and percentage of selected sociodemographic variables and knowledge level with self-care practice subscales. Finally, the results were summarized and presented in texts, figures, and tables.

## 4. Results and Discussion

### 4.1. Results

#### 4.1.1. Sociodemographic Characteristics of Hypertensive Patients

The response rate of the study was 100%. The median age of the participants was 56 years (SD ± 13.6). Most of the respondents, 178 (46.4%), were within the age group of 41–60 years. Majority of the respondents, 139 (36.2%), 266 (69.3%), and 255 (66.4%), were housewife, lived in urban area, and married, respectively. A large proportion of the study participants, 303 (78.9%), had no family history of hypertension (Table 1).

#### 4.1.2. Knowledge of Hypertensive Patients about Hypertension

The majority, 365 (92.4%) and 379 (98.7%), reported that hypertension is a serious disease and regular check-ups are important, respectively. But, 144 (37.5%) of the respondents were not aware about the normal BP level. Most of the respondents, 305 (79.4%) and 339 (88.3%), were aware of about the negative impact of smoking of cigarette and alcohol drinking, respectively. Around half of the respondents, 188 (49%), knew diets that consist of low-fat milk and whole wheat bread (Table 2).

**Figure 1:**
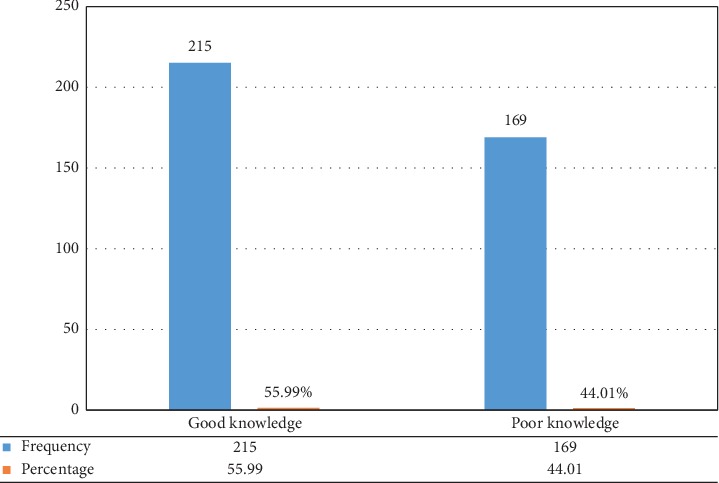
Knowledge level of the hypertensive patient, 2019.

Two hundred fifteen (56%) (95% CI: 51%, 60.7%) of participants scored median and above and considered having good knowledge towards hypertension, while 169 (44%) (95% CI: 39.3%, 49.0%) scored below the median and considered having poor knowledge (Figure 1).

#### 4.1.3. Self-Care Practice among the Respondents

Of the study participants, 228 (59.4%) (95% CI: 54.9%, 64.6%) had good self-care practice, while 156 (40.6%) (95% CI: 35.4%, 45.1%) had poor self-care practice. In terms of the six domains of self-care practice, 261 (68%), 266 (69.3%), and 81 (21.1%) had adherence to medication, low-salt diet, and physical activity, respectively (Table 3).

In the cross-tabulation of self-care practice with knowledge level, a higher frequency of good self-care practice was observed among those who had good hypertension knowledge.

### 4.2. Discussion

This study was intended to determine knowledge on hypertension and its self-care practice among adult hypertensive patients at the University of Gondar Comprehensive Specialized Hospital. Based on this aim, 56% and 59.4% had good knowledge and overall self-care practice, respectively.

More than half of hypertensive patients had good knowledge of hypertension in this study. This finding is similar to the study finding in South India (52.4%) [13]. On the other hand, it is higher than studies conducted in Nepal (14%) [14], Ethiopia (43.6%) [18], and Canada (18.2%) [15]. The possible reasons for the difference might be due to the study setting, sample size, and study period variation between the current study and previous studies.

In this study, almost sixty percent hypertensive patients had good self-care practice towards hypertension. This finding is higher than the studies conducted in Addis Ababa, Ethiopia (51.5%) [18] and south India (14%) [13]. These differences could be due to variation in residence and study period as majority of the participants were from urban in this study, which might help them to get information easily. In addition, it is the government's current agenda to work on noncommunicable diseases.

Similarly, in this study, majority of the participants had good medication adherence, which is lower than studies conducted in Saudi Arabia (83.7%) [11] and Western Nepal (85%) [21]. This variation might be due to the reason that majority of the participants in the current study were not knowledgeable about hypertension. In the current study, almost quadruple times of the respondents had low-salt diet adherence. This finding is lower than studies conducted in Saudi Arabia (79.3%) [11] and Ethiopia (94.6%) [19]. But, it is higher than the study conducted in Nigeria (18.5%) [22]. This might be due to the variation in the study setting, sample size, and study period variation. This study also revealed that below one-third of the participants had adhered with physical activity recommendations, which is lower than the study conducted in Saudi Arabia (57.3%) [11], but higher than study conducted in Nigeria (9.3%) [22]. The difference could be that participants were not well educated about self-care practice towards low-salt diet and physical activities in the current study. The current study pointed out that majority of the participants had quit smoking, which is an important aspect of self-care practice. This finding is in line with the study conducted in western Nepal (70%), and it is lower than the study conducted in Jimma, Ethiopia (98.5%) [19]. But, it is higher than the study conducted in Saudi Arabia (31%) [11]. This difference could be due to majority of the participants who were from urban in the current study, but the reverse is true in Saudi Arabia where life style and urbanization affect smoking condition. This study also ascertained that more than half of the study participants had good weight management practice, which is comparable to the study conducted in Saudi Arabia (59.9%) [11].

## 5. Clinical Relevance of the Study

As shown above and mentioned by different literatures, understanding the level of patients knowledge on hypertension and its self-care measures are an important topic to be addressed to fill treatment gaps. The current study points out that the patient's involvement in their own care can bring good clinical outcome. This will have an impact to avert hypertension-related complication and mortality.

## 6. Conclusions

The level of knowledge and self-care practice among hypertensive patients were relatively low, and almost 80% of participants had poor self-care practice in the physical activity domain. Hence, giving focus on awareness of hypertension and self-management of hypertensive patients are important. Furthermore, a large follow-up study is recommended.

## Figures and Tables

**Table 1:** Sociodemographic characteristics of hypertensive patients.

Variables	Frequency	Percent
*Age category (in years)*		
<40	53	13.8
40–60	178	46.4
61–80	143	37.2
≥81	10	2.6

*Sex*		
Male	184	47.9
Female	200	52.1

*Marital status*		
Single	31	8.1
Married	255	66.4
Divorced	33	8.6
Widowed	65	16.9

*Level of education*		
Illiterate	154	40.1
Primary education	114	29.7
Secondary education	54	14.1
College and above	62	16.1

*Occupation*		
House wife	139	36.2
Husband	56	14.6
Student	6	1.6
Merchant	55	14.3
Farmer	36	9.4
Government employee	50	13
NGO	31	8.1
Others^*∗*^	11	2.9

*Residence*		
Urban	266	69.3
Rural	118	30.7

*Income (in Birr)*		
≤500	33	8.6
501–1000	71	18.5
>1000	280	72.9

*Length since diagnosis (in years)*		
≤5	206	53.6
>5	178	46.4

*Family history*		
Yes	81	21.1
No	303	78.9

^*∗*^Others = labor worker, pension receiver, private teacher, driver, guard, private pharmacist, priest, and sanitation worker.

**Table 2:** Description of hypertension knowledge-related questions among hypertensive patients, 2019 (*n* = 384).

Questions	Frequency	Percent
Hypertension is a serious condition that can lead to complications		
Yes	365	92.4
No	29	7.6
An individual with hypertension should go for check-ups regularly		
Yes	379	98.7
No	5	1.3
It is important for a patient with hypertension to have a reliable means of blood pressure monitoring between visits to their health care provider		
Yes	370	96.4
No	14	3.6
A blood pressure level of above 130/90 is considered normal		
Yes	144	37.5
No	240	62.5
A blood pressure level of less than 120/80 is considered to be high		
Yes	154	40.1
No	230	59.9
Smoking cigarettes has a negative effect on persons with hypertension		
Yes	305	79.4
No	79	20.6
Drinking alcohol has a negative effect on persons with hypertension		
Yes	339	88.3
No	45	11.7
Increased physical exercise actually decreases the blood pressure of a person with hypertension		
Yes	322	83.9
No	62	16.1
A diet which contains fruits and vegetables is good for a person with hypertension		
Yes	301	78.4
No	83	21.6
A diet consisting of low-fat milk and whole wheat bread is good for a person with hypertension		
Yes	188	49
No	196	51
Corned beef and salted meat is good for a person with hypertension		
Yes	313	81.5
No	71	19.5
A meal rich in green bananas, baked chicken, and beans is good for a person with hypertension		
Yes	124	32.3
No	260	67.7

**Table 3:** Self-care practice level of hypertensive patients, 2019.

Self-care practice domains		Frequency	Percentage
Medication adherence	Adherent	261	68
	Not adherent	123	32

Low-salt diet	Adherent	266	69.3
	Not adherent	118	30.7

Physical activity	Adherent	81	21.1
	Not adherent	303	78.9

Smoking	Nonsmoker	272	70.8
	Smoker	112	29.2

Weight management	Good weight management practice	235	61.2
	Poor weight management practice	149	38.8

Alcohol	Abstainers	278	72.4
	Not abstainers	106	27.6

Overall self-care practice	Good	228	59.4
	Poor	156	40.6

## Data Availability

The datasets generated and/or analyzed during the current study are not publicly available due to some privacy reasons, but part of the raw dataset will be available from the corresponding author upon reasonable request.

## Additional Points

Since the study assessed the perceived patient's report, it might underestimate or overestimate the result.
